# Favorable function of Ectonucleoside triphosphate diphosphohydrolase 1 high expression in thyroid carcinoma

**DOI:** 10.1186/s41065-021-00198-6

**Published:** 2021-08-31

**Authors:** Jun-hua Luo, Yun-hua Zhu, Cheng Xiang

**Affiliations:** 1 Department of Thyroid and Breast Surgery, Yuhang Branch of the Second Affiliated Hospital of Zhejiang University, Hangzhou, 311100 Zhejiang China; 2 Department of Thyroid Surgery, Second Affiliated Hospital of Zhejiang University School of Medicine, Hangzhou, 310009 Zhejiang China

**Keywords:** ENTPD1, Thyroid carcinoma, High expression, Prognosis, Metabolic pathway

## Abstract

**Background:**

Ectonucleoside triphosphate diphosphohydrolase 1 (ENTPD1) has been proved to play a vital role in human cancers. Nevertheless, the exact role of ENTPD1 in thyroid carcinoma (THCA) remained unclear. This study aimed to evaluate its prognostic value and reveal the potential regulatory mechanism in THCA.

**Results:**

(1) Higher expression of ENTPD1 was found in THCA tissue compared with normal tissue (all *P* < 0.05). ENTPD1 expression was associated with age, sub-type and clinical stage of THCA patients (all *P* < 0.05). Immunohistochemistry showed its higher expression in patients with early stage. (2) ENTPD1 high expression was associated with favorable overall survival of THCA patients (all *P* < 0.05), especially for male patients and those with advanced stage, B-cells and Natural killer T-cells decreased (all *P* < 0.05). (3) Pathway analysis indicated that ENTPD1 mainly participated in metabolic process and negatively regulated metabolism-related pathway such as butanoate metabolism, pyruvate metabolism and fatty acid metabolism ((all *P* < 0.05). (4) ENTPD1 appeared genetic alteration in THCA, and the main mutation type of ENTPD1 was missense substitution (15.89%). (5) A weak correlation between ENTPD1 expression and methylation was found (*P* < 0.001). Methylation of ENTPD1 in THCA was lower than in normal group (*P* < 0.001), but it did not correlate with any clinical phenotypes of THCA patients.

**Conclusions:**

ENTPD1 was highly expressed in THCA, and ENTPD1 high expression contributed to the prognosis of THCA patients. The present study inferred that ENTPD1 might serve as a metabolism-related gene and play a critical role in THCA through regulating metabolic pathways.

## Background

Thyroid carcinoma (THCA) was one of the most common types of endocrine malignancy, and accounted for approximately 1.7% of total cancer diagnoses [1]. Among all the sub-types of THCA, papillary thyroid carcinoma (PTC) was the dominant form accounting for approximately 80%. Although patients with PTC showed the most favorable prognosis with 10-year survival over 95%, 5–20% of patients still faced the risk of disease recurrence and distant metastasis, which resulted in aggressive diseases and lethal outcomes [2]. Anaplastic thyroid carcinoma (ATC) was the most aggressive type with poor prognosis, and median overall survival (OS) was 11.9 months with 39% survival at 1 year [3]. Lymph node metastasis and extrathyroidal extension appeared in 40% of ATC patients, whereas the remaining 60% of patients displayed distant metastases [4]. Therefore, it was crucial to explore the fatal cancer's mechanism, find potential therapeutic target, improve patient prognosis and increase their survival time.

Ectonucleoside triphosphate diphosphohydrolase 1 (ENTPD1), also known as CD39, was a rate-limiting enzyme in the generation of immunosuppressive adenosine [5]. ENTPD1 was an integral membrane protein that metabolized adenosine diphosphate (ADP) and extracellular adenosine triphosphate (ATP) to adenosine monophosphate (AMP) [6]. It was secreted by B cells, dendritic cells, T cells and neoplastic cells in a number of hematologic malignancies [5]. Currently, growing evidences have described that ENTPD over-expression and dys-regulation were associated with human cancers. It was demonstrated that ENTPD1 high expression positively correlated with tumor stage in squamous cell carcinoma of the head and neck, and led to an inferior patients’ overall survival [7]. ENTPD1 expression can inhibit natural killer (NK) cell activity and be permissive for the growth of metastatic tumors in the liver [8]. Another study indicated that ENTPD1 was abundantly expressed in liver metastases and tumor draining lymph nodes from metastatic rectal adenocarcinoma [9]. However, the study surprisingly found that patients with higher ENTPD1 density in tumor cells were more likely to have favorable characteristics (early TNM and N stages) and better overall survival. It followed that ENTPD1 expression and its prognosis significance were discrepant among different cancer types. To our knowledge, the potential role of ENTPD1 in THCA has not been revealed and remained unclear.

In this study, we first evaluated the expression of ENTPD1 in THCA, as well as assessed the significance of ENTPD1 expression on the clinical outcome of THCA patients. Furthermore, we explored the significant ENTPD1-associated pathways involved in THCA. We also evaluated the the genetic alteration and methylation level of ENTPD1 in THCA patients. This study will provide a new insight into the molecular mechanisms of thyroid carcinoma.

## Results

### mRNA expression of ENTPD1 in THCA

**Fig. 1:**
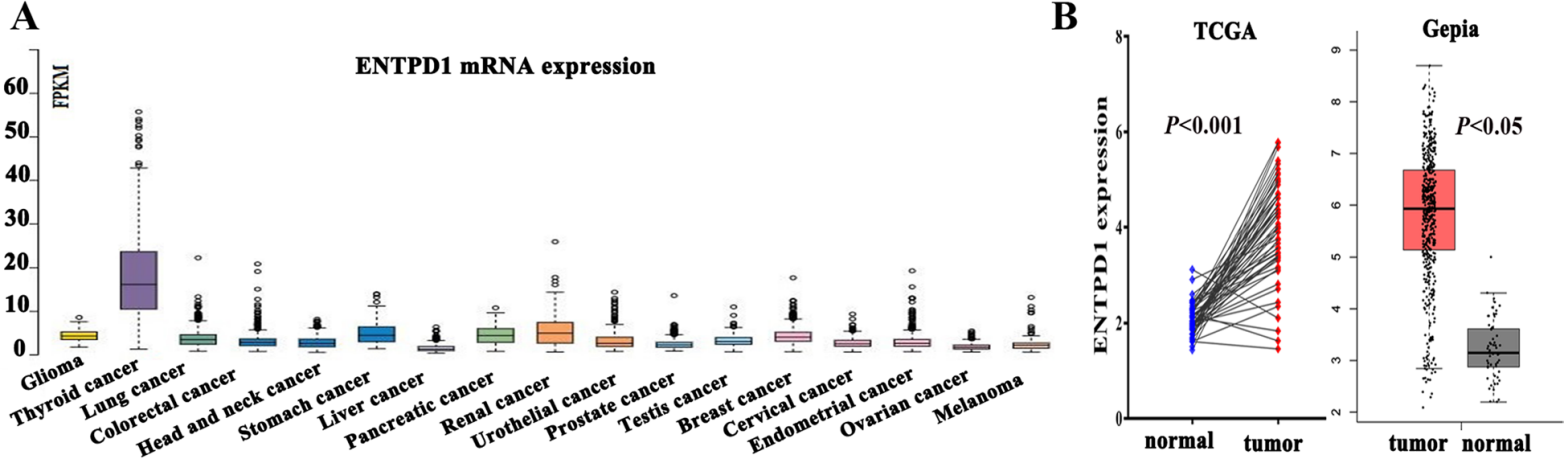
ENTPD1 mRNA expression. **A** Various cancers. The data was obtained from HPA database. **B** Differential expression in THCA and normal tissues. The data was obtained from TCGA and Gepia database

The expression of ENTPD1 mRNA in human cancers was firstly evaluated. As observed in Fig. 1A, the expression of ENTPD1 was relatively higher in thyroid cancer than in other cancers. Quantitative analysis in Fig. 1B showed that ENTPD1 was highly expressed in THCA tissue than in normal tissue (all *P* < 0.05).

**Fig. 2:**
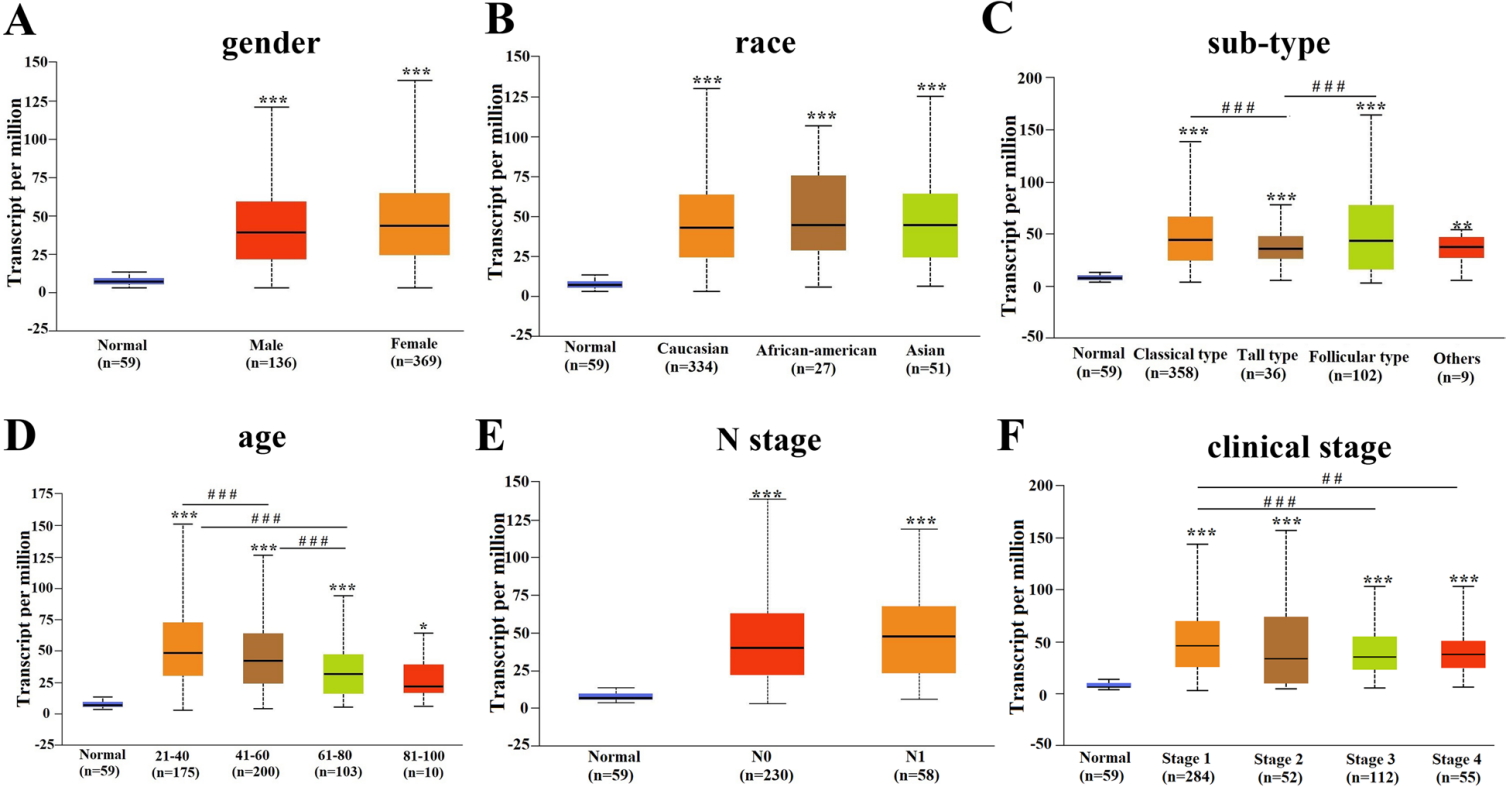
ENTPD1 mRNA expression based on clinical characteristics. **A** Gender. **B** Race. **C** Sub-type. **D** Age. **E** N stage; **F** Clinical stage. Compared with normal group: ^*^P < 0.05, ^**^P < 0.01 and ^***^P < 0.001. Multiple comparisons: ^##^*P* < 0.01 and ^###^*P* < 0.001. The data was obtained from UALCAN database which was based on TCGA data

For better elucidating the role of ENTPD1 in THCA, the expression of ENTPD1 was identified under different clinical parameters. Our result showed that ENTPD1 was highly expressed in THCA tissue compared with the normal group (all *P* < 0.05). The expression of ENTPD1 did not correlate with gender (Fig. 2A) and race (Fig. 2B) of THCA patients. The sub-type of THCA patients influenced the ENTPD1 expression, and higher expression was observed in classical and follicular thyroid papillary carcinoma (Fig. 2C). The negative correlation between age and ENTPD1 expression was presented in THCA patients, and higher expression appeared in patients with young age (Fig. 2D). There was no statistical significance of ENTPD1 expression based on N stage (Fig. 2E). However, clinical stage of THCA patients was statistically associated with ENTPD1 expression, and patients in stage 1 showed higher expression of ENTPD1 (Fig. 2F). Totally, the expression of ENTPD1 correlated with the age, sub-type and clinical stage of THCA patients.

### Protein expression of ENTPD1 in THCA

**Fig. 3:**
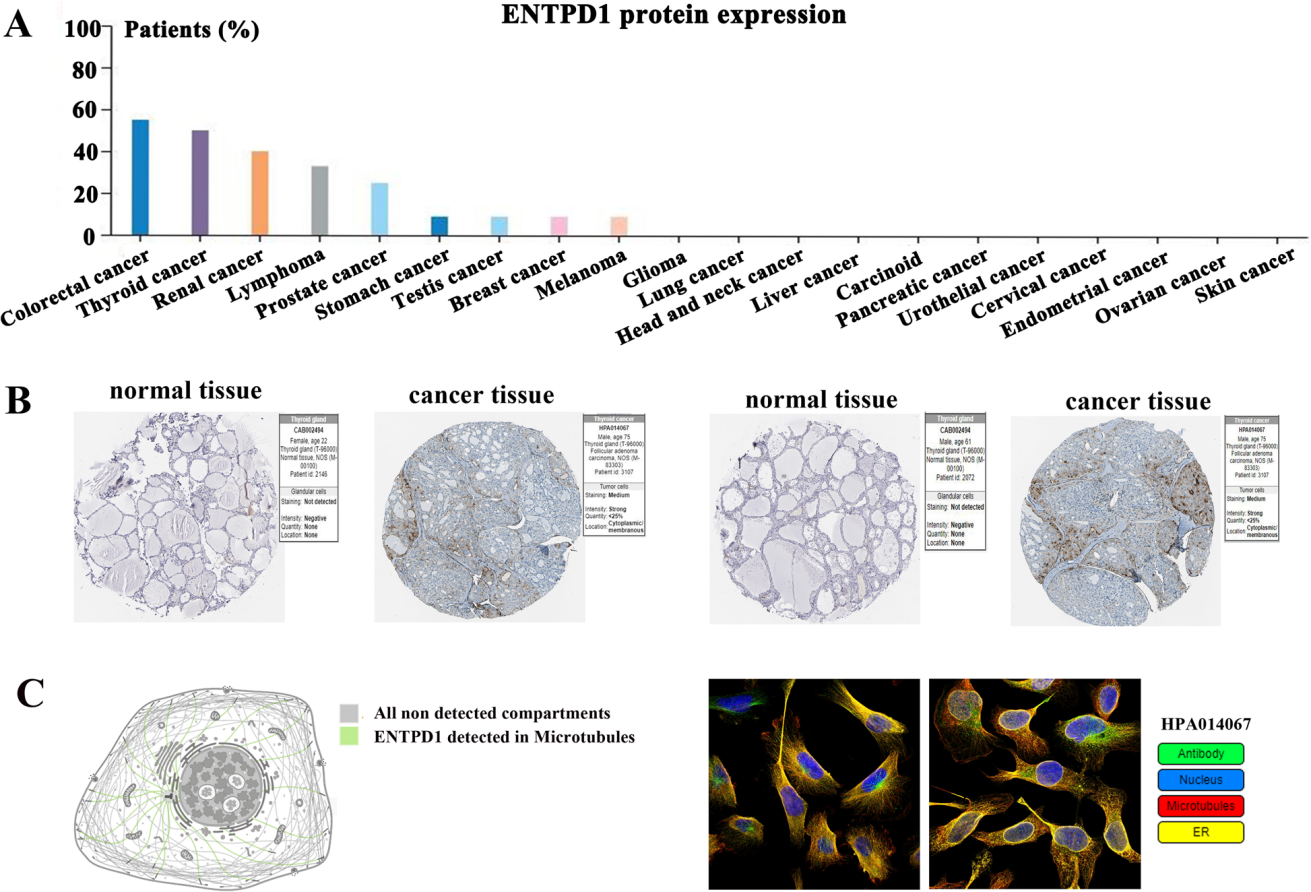
ENTPD1 protein expression. **A** Expression in various cancers. **B** Representative immunohistochemistry images of ENTPD1 protein in THCA and normal tissues. **C** ENTPD1 protein location within cancer cell. All data were obtained from HPA database

We then evaluated the protein expression of ENTPD1 in cancer tissues. As presented in Fig. 3A, most of cases in thyroid cancer showed the high expression of ENTPD1 protein. The immunohistochemistry images (Fig. 3B) showed that ENTPD1 staining was not detected in normal tissue, and protein intensity was negative. However, medium staining and strong intensity of ENTPD1 protein expression was observed in thyroid cancer tissue. Figure 3C disclosed the protein location of ENTPD1 in cancer cells, and suggesting that ENTPD1 was mainly localized to the microtubules.

**Fig. 4:**
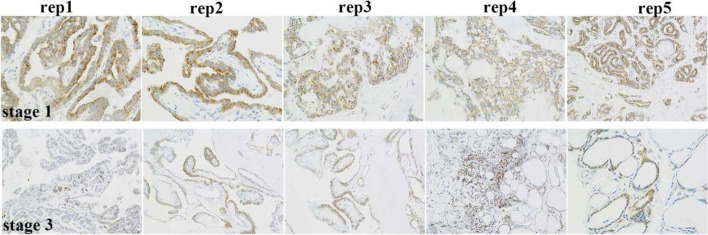
The expression verification of ENTPD1 by immunohistochemistry in stage 1 and stage 3 patients

The above results indicated that ENTPD1 mRNA and protein were highly expressed in THCA patients compared with normal group, and we then performed the immunohistochemistry to confirm the detailed ENTPD1 expression among THCA patients. The Fig. 4 confirmed that ENTPD1 expression in stage 1 was higher than in stage 3, suggesting its higher expression in early stage in THCA patients.

### Prognostic value of ENTPD1 in THCA

**Fig. 5:**
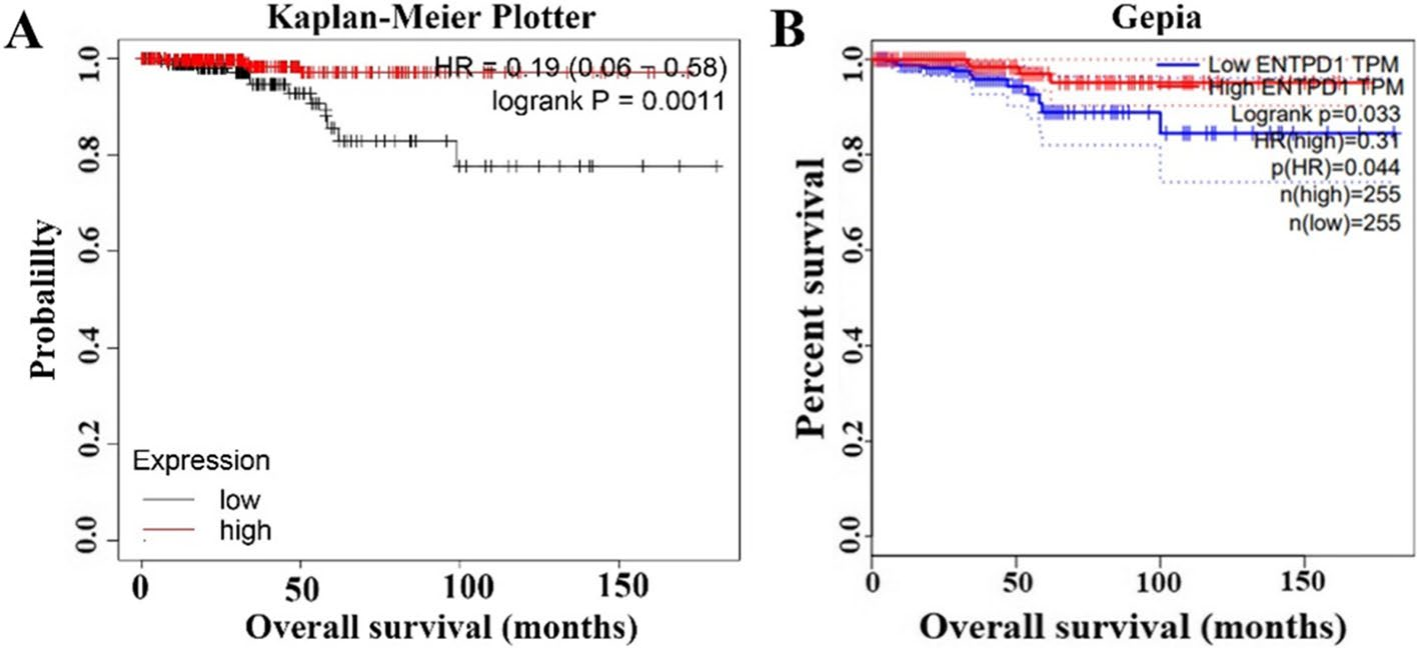
The influence of ENTPD1 expression on the overall survival of patients. **A** Kaplan–Meier Plotter database. **B** Gepia database

Clinical outcome was the common concern of clinicians and patients, and we further determined whether ENTPD1 expression influenced the clinical outcome of THCA patients. Kaplan–Meier Plotter database indicated that ENTPD1 high expression prolonged the survival time of patients (Fig. 5A  , *P*= 0.0011). Gepia database also confirmed that high expression of ENTPD1 statistically increased the overall survival time of patients with THCA (Fig. 5B , *P*= 0.033). As indicated above, ENTPD1 was highly expressed in THCA and high expression favorably improved the prognosis of patients.

We further performed the restrict survival analysis to explore the effects of ENTPD1 expression on the prognosis of THCA patients. Table 1 showed that ENTPD1 expression was statistically associated with the survival of male patients (*P* < 0.001). The survival between ENTPD1 high and low expression groups was statistically significant in stage 3 (*P* = 0.0085) and stage 4 (*P* = 0.0038) patients. It followed that ENTPD1 did not affect the survival of female patients and those in early cancer stages. The mutation burden did not influence the survival of patients with ENTPD1 high/low expression. Regardless of the enriched/decreased macrophages and regulatory T-cells, the survival difference was observed among patients with ENTPD1 high and low expression (all *P* < 0.05). Moreover, the survival time was different between ENTPD1 high and low expression groups in terms of decreased B-cells and Natural killer T-cells (all *P* < 0.05).

**Table 1:** The influence of ENTPD1 expression on the survival of THCA patients

Characteristics	Subtypes	HR (95%CI)	Logrank *P*-value
Gender	female	0.34 (0.09–1.19)	0.076
	male	0.04 (0–0.31)	**7.9E-06**
Cancer stage	1	2.41 (0.15–38.52)	0.520
	2	0.30 (0.02–4.87)	0.370
	3	0.15 (0.03–0.76)	**8.5E-03**
	4	0.08 (0.01–0.73)	**3.8E-03**
Mutation burden	high	0.25 (0.06–0.95)	**0.028**
	low	0.12 (0.01–1.20)	**0.032**
B-cells	enriched	0.22 (0.02–2.10)	0.150
	decreased	0.12 (0.03–0.43)	**1.0E-04**
Macrophages	enriched	0.16 (0.03–0.80)	**0.011**
	decreased	0.19 (0.04–0.93)	**0.021**
Natural killer T-cells	enriched	0.39 (0.07–2.14)	0.260
	decreased	0.08 (0.02–0.37)	**3.3E-05**
Regulatory T-cells	enriched	0.21 (0.07–0.65)	**2.7E-03**
	decreased	0 (0-Inf)	**0.049**

*Abbreviation*: *HR* Hazard ratio. *CI* Confidence interval

### Functional enrichment and GSEA analyses

In order to disclose the function of ENTPD1 in THCA, this study first determined the co-expressed genes with ENTPD1 in THCA. The top 200 genes were finally selected according to the absolute value of spearman's correlation and *P* < 0.001. GO annotation was then performed to explore the biological function of co-expressed genes.

**Fig. 6:**
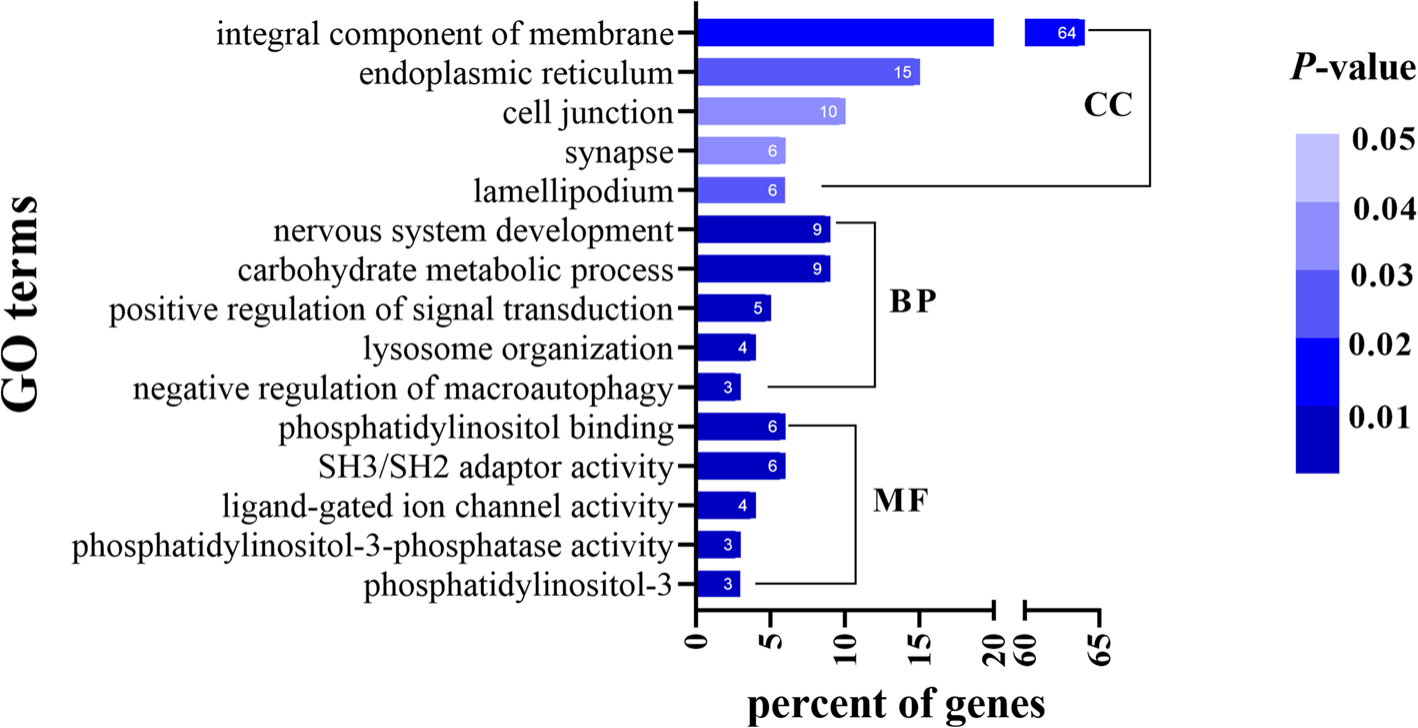
GO annotation analysis on co-expressed genes

The top5 GO terms were shown in Fig. 6. In terms of molecular function (MF), co-expressed genes were mainly enriched in phosphatidylinositol 3, phosphatidylinositol-3-phosphatase activity, ligand-gated ion channel, SH3/SH2 adaptor activity, phosphatidylinositol binding. In biological process (BP) category, these co-expressed genes were mainly associated with nervous system development and carbohydrate metabolic process. In addition, cellular component (CC) analysis showed that these genes were primarily accumulated in the integral component of membrane and endoplasmic reticulum.

This study also investigated the significant pathways associated with co-expressed genes, and KEGG pathway enrichment analysis was performed using WebGestal and KOBAS databases. The top 5 consistent pathways were presented in Table 2. KEGG analysis indicated that co-expressed genes were significantly associated with metabolism-related pathways, including fat digestion and absorption, glycerophospholipid metabolism and ether lipid metabolism. In addition, pathway of transcriptional misregulation in cancer was also detected.

**Table 2:** The KEGG pathway analysis on co-expressed genes

Pathway ID and name	*P*-value		
	WebGestalt	KOBAS	
hsa01100: Metabolic pathways	4.81E-05		1.78E-02
hsa05202: Transcriptional misregulation in cancer	1.13E-02		4.40E-04
hsa04975: Fat digestion and absorption	8.18E-03		1.42E-03
hsa00564: Glycerophospholipid metabolism	1.68E-02		1.72E-03
hsa00565: Ether lipid metabolism	1.19E-02		2.05E-03

**Fig. 7:**
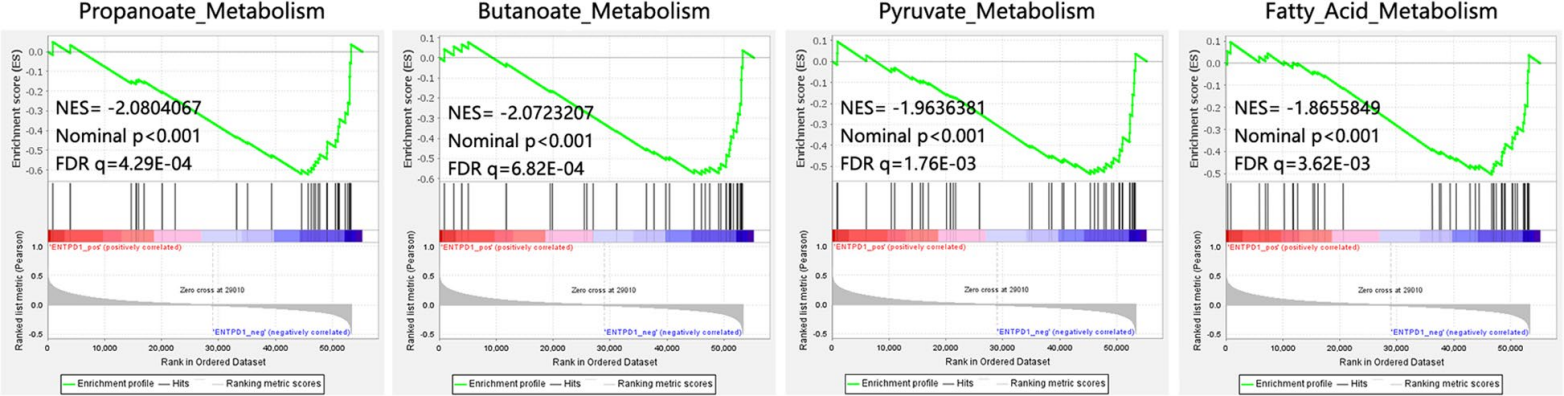
The GSEA analysis on the ENTPD1 involved in THCA

We also conducted the GSEA analysis to reveal the potential role of ENTPD1 involved in THCA. In addition to the metabolism-related pathways detected above, GSEA analysis (Fig. 7) also found that ENTPD1 was negatively associated with other metabolic pathways such as propanoate metabolism, butanoate metabolism, pyruvate metabolism, fatty acid metabolism. We speculated that ENTPD1 might influence the progression of THCA through regulating metabolic pathways.

**Fig. 8:**
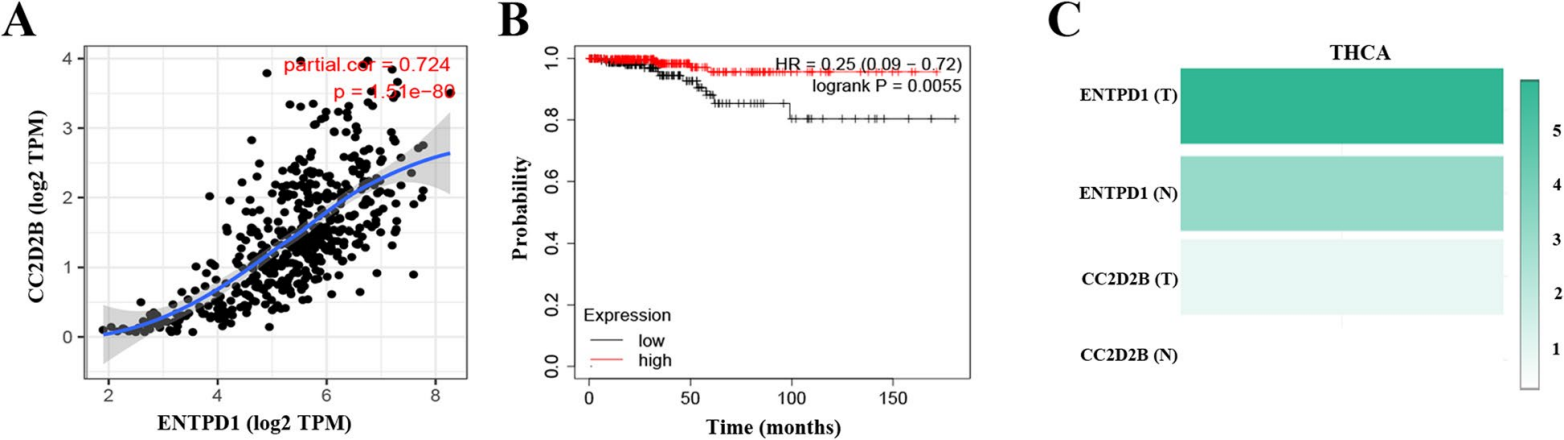
Expression analysis on CC2D2B. **A** Correlation of ENTPD1 with CC2D2B. **B** Prognosis analysis on CC2D2B in THCA. **C** Expression comparison. (T: tumor; N: normal). The data was obtained from Gepia

Among the whole co-expressed genes, CC2D2B showed the highest correlation coefficient with ENTPD1. Figure 8A further confirmed the strong correlation between them (*P* < 0.001). High expression of CC2D2B was positively associated with the overall survival of THCA patients (Fig. 8B, HR = 0.25, *P* = 0.0055). Figure 8C presented that CC2D2B expression was higher in THCA than in normal tissue. Additionally, CC2D2B expression was lower than the expression of ENTPD1 among THCA patients.

### Methylation analysis of ENTPD1 in THCA

**Fig. 9:**
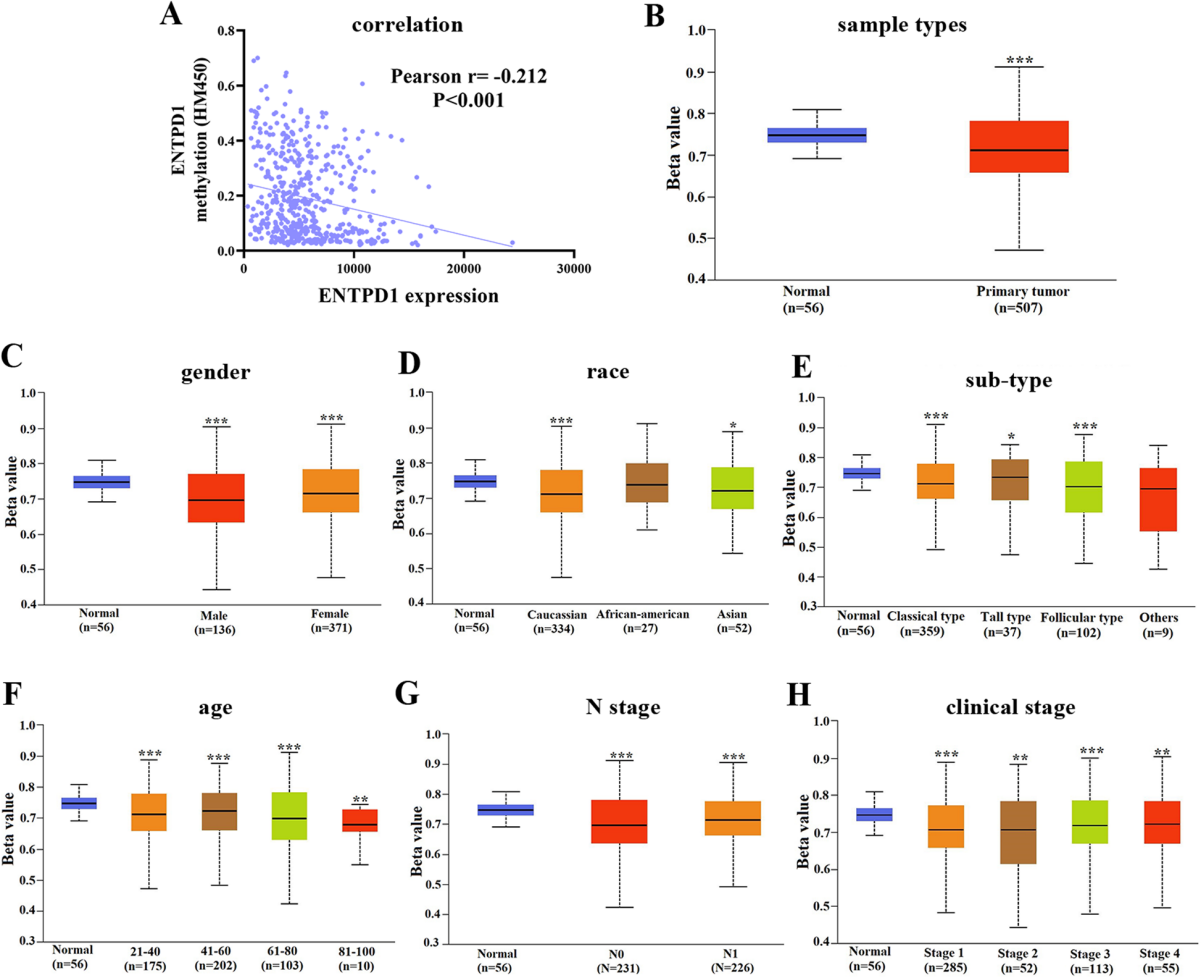
ENTPD1 methylation analysis. **A** correlation analysis between ENTPD1 methylation and expression; **B** sample type; **C** gender; **D** race; **E** sub-type; **F** age; **G** N stage; **H** clinical stage. The Beta value indicated level of DNA methylation ranging from 0 (unmethylated) to 1 (fully methylated). Compared with normal group: ^*^P < 0.05, ^**^P < 0.01 and ^***^P < 0.001. There was no difference of methylation degree among any groups in THCA patients after multiple comparisons. The data was obtained from UALCAN database

DNA methylation was an important event in the early stage of tumorigenesis, and promoter methylation could regulate the gene expression at the transcriptional level. Our result revealed the weak association between ENTPD1 expression and methylation (Fig. 9A , *P*< 0.001). ENTPD1 methylation was obviously lower in primary tumor than the normal tissue (Fig. 9B , *P*< 0.001). Although patients with male, caucasian, follicular type, N0 stage, age (81–100) and clinical stage (1, 2) showed the lowest methylation (Figs.9C-H), multiple-comparison found no difference of ENTPD1 methylation in THCA patients base on variant phenotypes.

**Fig. 10:**
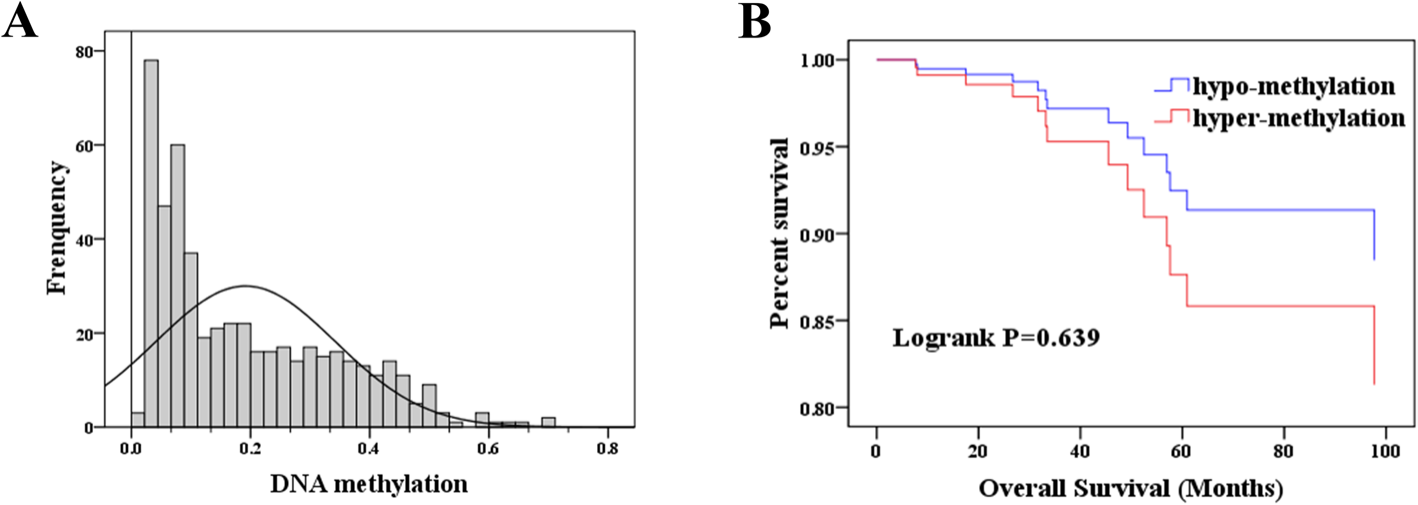
ENTPD1 methylation and prognostic value assessment. **A** Distribution map of ENTPD1 methylation degree. X-axis represented the degree of methylation (beta value) and Y-axis represented the frequency. **B** Survival curve of patients. hypo-methylation: beta value < 0.3; hyper-methylation: beta value > 0.5

As seen from frequency distribution of methylation level, most of THCA patients showed ENTPD1 hypo-methylation (Fig. 10A). Survival analysis indicated that hypo-methylation increased the survival time of THCA patients, but statistical significance was not observed compared with hyper-methylation groups (Fig. 10B).

### Genetic alteration analysis of ENTPD1 in THCA

**Fig. 11:**
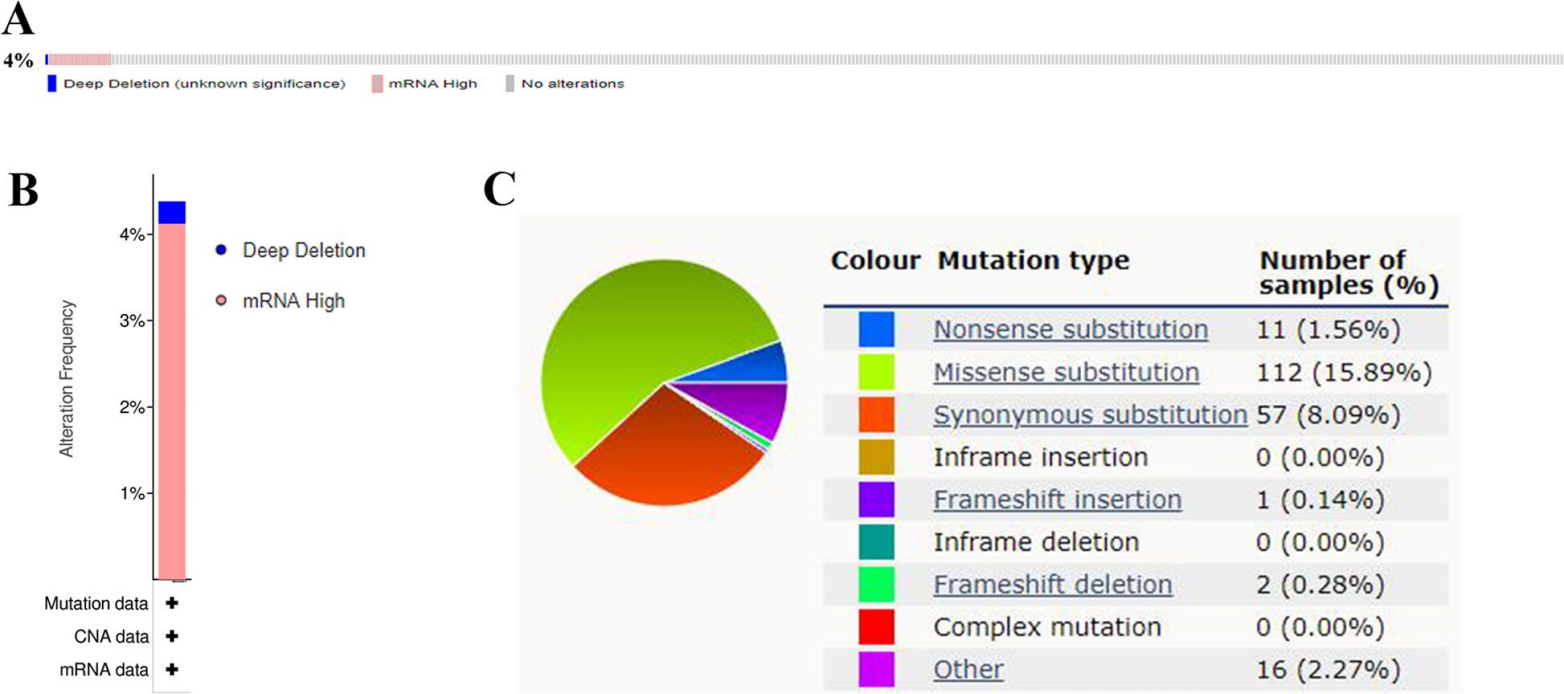
The genetic alteration of ENTPD1 in thyroid carcinoma. **A** Mutation frequency. **B** Alteration frequency in patients with papillary thyroid carcinoma. The data of A and B were obtained from cBioportal database. **C** Mutation type. The data was obtained from COSMIC database

The genetic alteration of gene played an important role in the progression of cancers. We used 388 samples with papillary thyroid carcinoma in cBioPortal database to explore the genetic alteration of ENTPD1. Among patients, 4% of subjects appeared ENTPD1 mutation (Fig. 11A), and principal manifestation of genetic alteration was mRNA high (Fig. 11B). The main mutation type of ENTPD1 in cancers was missense substitution with 15.89% (Fig. 11C).

## Discussion

ENTPD1 was a critical member of ectonuclotidases family. Over-expression of ENTPD1 has been shown in various cancers, which adversely affected the clinical outcome of patients. The study [10] found that IL-6 could induce ENTPD1 expression on tumor-infiltrating NK cells, which predicted a poor prognosis in esophageal squamous cell carcinoma. ENTPD1 was also expressed at higher rate in gastric cancer tissue, and over-expression correlated with poor overall survival [11]. But so far there was no study revealed the potential effects of ENTPD1 in THCA. Therefore, current study initially evaluated the expression of ENTPD1 in THCA, and assessed its prognostic significance through bioinformatics analysis.

Our results showed that ENTPD1 was highly expressed in THCA. The expression of ENTPD1 was associated with age, sub-type and clinical stage of THCA patients. The interesting thing was that ENTPD1 high expression contributed to the survival of THCA patients and achieved a good prognosis. However, another research revealed the favorable function of ENTPD1 expression in process of cancers. It has demonstrated that adult T-cell leukemia/lymphoma (ATLL) cells expressed ENTPD1 at a high rate, which contributed ATLL cells to escape anti-tumor immunity through the extracellular ATPDase-Adenosine cascade [12]. Other study [13] indicated that ENTPD1 was up-regulated in cytarabine (AraC)-resistant leukemic cells, and ENTPD1 high activity promoted AraC resistance by enhancing mitochondrial activity through activation of a cAMP-mediated adaptive mitochondrial stress response. It appeared that ENTPD1 played a vital role in different types of cancers.

To further elucidate the potential function of ENTPD1, we explored the co-expressed genes with ENTPD1 in THCA and then performed functional enrichment analysis. Enrichment analysis indicated that ENTPD1 significantly participated in the metabolism-related pathway, and mainly played catalytic and binding activities. ENTPD1 has been proved to participate in glucose metabolism in THCA as well. Glucose metabolic profiles were intricately associated with tumor differentiation in THCA, and high glycolysis signature significantly resulted in poor prognosis [14]. Other research [15] found that ENTPD1 was a modulator of extracellular nucleotide signaling and influenced metabolism, deletion of ENTPD1 both directly and indirectly impacted insulin regulation and hepatic glucose metabolism. As indicated in our results and previous research, ENTPD1 might be acted as a metabolism-associated gene and regulate the metabolic pathways in THCA. Other metabolic genes in THCA were also discovered such as LPCAT2, HSD17B8, ACOT7 and PDE8B [16]. KMT5A was found to modulate lipid metabolism of PTC in *vitro* as well [17]. The THCA might be regulated by metabolic genes through regulating certain metabolism process. Not insignificant, however, was that metabolism-related disease such as insulin resistance, dysglycemia, high BMI and hypertension, can significantly increase the thyroid cancer risk [18].

In addition, we also explored the influence of clinical phenotypes on ENTPD1 methylation degree. We found weak correlation between ENTPD1 expression and methylation. The methylation degree of ENTPD1 in THCA tissue was lower than normal group. It seemed that higher ENTPD1 expression has favorable characteristics (early clinical stage and young age). However, ENTPD1 methylation was unrelated to any clinical phenotypes of THCA patients.

It followed that ENTPD1 might play a vital role in thyroid carcinoma through regulating important pathways. Our study indicated the ENTPD1 as a significant biomarker involved in thyroid carcinoma. If ENTPD1 can be used in the pre-operative diagnosis of thyroid carcinoma, it will become a promising assist to find out more precise and efficient way to solve the patients' problem. At present, the presence of micropapillary tumors can be predicted preoperatively by ultrasound-guided fine-needle aspiration biopsy (FNAB) in papillary thyroid carcinoma [19]. Although the examination of samples collected from lymph nodes by fine-needle aspiration biopsy cytology (FNAB-C) was extremely specific for the diagnosis of metastases, its sensitivity is low, especially in paucicellular samples [20]. Therefore, if ENTPD1 can be able to aid the FNAB diagnosis, it will be a useful biomarker for benefiting patients.

Although we clarified the significance of ENTPD1 in THCA, the limitation should be acknowledged regarding this study. Current study mainly analyzed the potential function of ENTPD1, while the value for increasing the diagnostic accuracy needed further study. Whether ENTPD1 was related to glucose metabolism in THCA should be further determined. In addition, the effects caused by CC2D2B and ENTPD1 needed further revelation.

## Conclusion

The present study aimed at clarifying the critical role of ENTPD1 in THCA via bioinformatics analysis. The high expression of ENTPD1 was observed in THCA, and ENTPD1 high expression was beneficial to the prognosis of THCA patients. ENTPD1 possibly involved in THCA through negatively regulating metabolism-related pathway, which indicated that ENTPD1 might serve as a potential metabolic gene in THCA. Our results provided a new insight into the exploration of pathogenesis and molecular biomarker for THCA.

## Methods

### mRNA expression analysis of ENTPD1

The expression of ENTPD1 mRNA in human cancers was explored via Human Protein Atlas (HPA) database (https://www.proteinatlas.org/). Differential expression of ENTPD1 in THCA and normal tissues was further determined through Gepia (http://gepia.cancer-pku.cn/) and TCGA databases. In addition, the association of ENTPD1 expression with clinical features of THCA patients was evaluated with UALCAN database (http://ualcan.path.uab.edu/). The clinical features included histological sub-type, race, gender, age, clinical stage and nodal metastasis status of patients.

### Protein expression analysis of ENTPD1

Protein expression of ENTPD1 in human cancers, differential expression in THCA and normal tissues, and protein location in cancer cell, were also assessed in HPA database. HPA database aims to map all the human proteins in cells, tissues and organs using an integration of various omics technologies, including antibody-based imaging, mass spectrometry-based proteomics, transcriptomics and systems biology. In addition, the protein expression difference in THCA patients under stage 1 and stage 3 was further verified in this study by immunohistochemistry.

### Prognosis analysis on ENTPD1

The potential influence of ENTPD1 expression on the prognosis of THCA patients was also estimated. Kaplan–Meier Plotter (http://kmplot.com/) and Gepia databases were applied to assess the influence of ENTPD1 expression on the overall survival of patients. The patients split method in Kaplan–Meier Plotter was set as auto select best cutoff. The median of ENTPD1 expression was set as the group cutoff in Gepia database. Subsequently, the restrict survival analysis between ENTPD1 high and low expression groups was performed in terms of gender, clinical cancer stages, mutation burden, B-cells, macrophages, natural killer T-cells and regulatory T-cells.

### Functional enrichment and GSEA analysis

For better revealing the function of ENTPD1 in THCA, the co-expressed genes with ENTPD1 were obtained through cBioportal database (http://www.cbioportal.org/). A total of 20,095 genes were found, of which the top 200 genes were finally selected according to absolute spearman's correlation and threshold of *P*-value less than 0.001. We then performed GO analysis to annotate co-expressed genes using online tool of DAVID (https://david.ncifcrf.gov/), and their function can be classified by biological processes (BP), molecular Function (MF) and cellular component (CC). KEGG pathway analysis was applied to dispose biological pathways related to co-expressed genes by KOBAS (http://kobas.cbi.pku.edu.cn/) and WebGestalt (http://www.webgestalt.org/) databases. GSEA pathway analysis was subsequently performed to reveal the potential mechanism associated with ENTPD1 involved in THCA. Threshold value was set as *P* < 0.05.

### Methylation analysis of ENTPD1

DNA methylation closely correlated with the gene expression, and may be one of the mechanisms of disease progression. Therefore, we assessed the association of DNA methylation with gene expression and clinical features. DNA methylation profile of thyroid cancer samples was retrieved from cBioportal database (http://www.cbioportal.org/). Pearson correlation analysis was conducted to assess association between ENTPD1 expression and methylation. ENTPD1 promoter methylation profiles based on sample type, cancer type, race, gender, age, clinical stage and metastasis status were visualized with UALCAN database. The Beta value indicated level of DNA methylation ranging from 0 (unmethylated) to 1 (fully methylated). Different beta value cut-off has been considered to indicate hyper-methylation (Beta value > 0.5) or hypo-methylation (Beta-value < 0.3). In addition, the distribution of methylation of ENTPD1 was evaluated, and influence on OS was assessed between hyper- and hypo-methylation groups.

### Genetic alteration analysis

The cBioPortal database was also used to explore the genetic alteration of ENTPD1 in thyroid cancer. As the main subtypes of THCA, we selected 388 samples with papillary thyroid carcinoma to assess the genetic alteration of ENTPD1. The mutation frequency of ENTPD1 among patients can be obtained via cBioPortal database. The mutation type of ENTPD1 was subsequently explored through COSMIC database (https://cancer.sanger.ac.uk/cosmic).

### Statistical analysis

Statistical software SPSS 19.0 was used for analyzing the clinical data. T test was used when the data satisfied homogeneity variance and normal distribution. Otherwise, Mann Whitney U test was applied for assessing difference. Kruskal–Wallis H test and Bonferroni's post-hoc test were performed for multiple comparisons. *P* < 0.05 was regarded as statistical significance.

## Data Availability

The datasets used and/or analysed during the current study are available from the corresponding author on reasonable request.

## Abbreviations

ATC: Anaplastic thyroid carcinoma
BP: Biological processes
CC: Cellular component
ENTPD1: Ectonucleoside triphosphate diphosphohydrolase 1
GO: Gene ontology
KEGG: Kyoto Encyclopedia of Genes and Genomes
MF: Molecular Function
OS: Overall survival
PPI: Protein–protein interaction
PTC: Papillary thyroid carcinoma
THCA: Thyroid carcinoma
